# The *Coxiella burnetii* T4SS Effector AnkF Is Important for Intracellular Replication

**DOI:** 10.3389/fcimb.2020.559915

**Published:** 2020-11-13

**Authors:** Julian Pechstein, Jan Schulze-Luehrmann, Stephanie Bisle, Franck Cantet, Paul A. Beare, Martha Ölke, Matteo Bonazzi, Christian Berens, Anja Lührmann

**Affiliations:** ^1^ Mikrobiologisches Institut-Klinische Mikrobiologie, Immunologie und Hygiene, Universitätsklinikum Erlangen, Friedrich-Alexander-Universität Erlangen-Nürnberg, Erlangen, Germany; ^2^ Institut de Recherche en Infectiologie de Montpellier (IRIM), Centre National de la Recherche Scientifique (CNRS), Université de Montpellier, Montpellier, France; ^3^ Coxiella Pathogenesis Section, Laboratory of Bacteriology, Rocky Mountain Laboratories, National Institute of Allergy and Infectious Diseases, National Institutes of Health, Hamilton, MT, United States; ^4^ Friedrich-Loeffler-Institut, Institut für Molekulare Pathogenese, Jena, Germany

**Keywords:** Q fever, type IV secretion system, effector protein, vimentin, phagosome, *Coxiella burnetii*

## Abstract

*Coxiella burnetii* is an obligate intracellular pathogen and the causative agent of the zoonotic disease Q fever. Following uptake by alveolar macrophages, the pathogen replicates in an acidic phagolysosomal vacuole, the *C. burnetii*-containing vacuole (CCV). Effector proteins translocated into the host cell by the type IV secretion system (T4SS) are important for the establishment of the CCV. Here we focus on the effector protein AnkF and its role in establishing the CCV. The *C. burnetii* AnkF knock out mutant invades host cells as efficiently as wild-type *C. burnetii*, but this mutant is hampered in its ability to replicate intracellularly, indicating that AnkF might be involved in the development of a replicative CCV. To unravel the underlying reason(s), we searched for AnkF interactors in host cells and identified vimentin through a yeast two-hybrid approach. While AnkF does not alter vimentin expression at the mRNA or protein levels, the presence of AnkF results in structural reorganization and vesicular co-localization with recombinant vimentin. Ectopically expressed AnkF partially accumulates around the established CCV and endogenous vimentin is recruited to the CCV in a time-dependent manner, suggesting that AnkF might attract vimentin to the CCV. However, knocking-down endogenous vimentin does not affect intracellular replication of *C. burnetii*. Other cytoskeletal components are recruited to the CCV and might compensate for the lack of vimentin. Taken together, AnkF is essential for the establishment of the replicative CCV, however, its mode of action is still elusive.

## Introduction

The obligate intracellular Gram-negative pathogen *Coxiella burnetii* is the causative agent of the zoonotic disease Q fever (Maurin and Raoult, 1999). With the exception of New Zealand, *C. burnetii* is distributed worldwide. The bacterium can infect a vast range of species (Maurin and Raoult, 1999; Gonzalez-Barrio and Ruiz-Fons, 2019), but live-stock animals, such as cattle, sheep, and goats, are the most important natural reservoirs and also the main source of human infections (Rodolakis, 2009). An acute infection might be symptom-free or cause a flu-like illness (Maurin and Raoult, 1999). The development of pneumonia or granulomatous hepatitis are also common symptoms of acute Q fever (Raoult et al., 2005). Immunocompromised people with preceding cardiac valve pathology and pregnant women are mainly at risk of developing chronic Q fever. Its typical symptoms include endocarditis and vascular infections (Maurin and Raoult, 1999). While good treatment options are available for acute Q fever, they are missing for chronic Q fever. Thus, chronic Q fever is treated by a combination of doxycycline and hydroxychloroquine for at least 18 months (Kersh, 2013). This lengthy treatment comes with severe side effects and, as a consequence, limited compliance.

Primary infection in humans occurs in alveolar macrophages after inhalation of *C. burnetii*-contaminated dust particles or aerosols (Khavkin and Tabibzadeh, 1988). However, non-phagocytic cells are also susceptible to infection (Voth and Heinzen, 2007). Host cell invasion of professional phagocytes by *C. burnetii* involves α_v_β_3_ integrin receptors and actin-dependent membrane ruffling (Baca et al., 1993; Capo et al., 1999; Dellacasagrande et al., 2000; Aguilera et al., 2009). In non-professional phagocytes, the bacterial invasin OmpA and cortactin are involved (Rosales et al., 2012; Martinez et al., 2014). Following internalization, the bacteria reside within the *C. burnetii*-containing vacuole (CCV), which develops into a phagolysosomal-like compartment with an acidic pH (van Schaik et al., 2013). Most bacteria are killed under these conditions, but for *C. burnetii* they are optimal for proliferation (Hackstadt and Williams, 1981). Moreover, expression and activity of the type IV secretion system (T4SS) is enabled under acidic conditions (Coleman et al., 2004; Newton et al., 2013). The fact that bacteria lacking the T4SS are unable to replicate intracellularly (Carey et al., 2011) demonstrates that the T4SS is a major virulence determinant. It is used to inject virulence factors, so-called effector proteins, which allows reprograming of the host cell for the benefit of the pathogen (Lührmann et al., 2017). Translocation of effector proteins starts around 8 h post-infection and translocation rates increase in a time-dependent manner (Newton et al., 2013). Several of the ~150 identified effector proteins interfere with vesicular trafficking or localize to the CCV membrane. The activity of T4SS effector proteins allows the massive expansion of the CCV, which can occupy the majority of the host cell´s volume (Lührmann et al., 2017). How *C. burnetii* ensures the stability of this huge compartment is not understood, but galectins (Mansilla Pareja et al., 2017) and actin (Colonne et al., 2016; Miller et al., 2018) might be involved.

Here we report that the T4SS effector protein AnkF (CBU0447) is important for optimal intracellular replication of *C. burnetii*. Ectopically expressed AnkF localized throughout the cytosol, the nucleus and partially around the CCV. It also interacted with the intermediate filament vimentin, which is recruited to the CCV in a time-dependent manner. However, siRNA-mediated knock-down of vimentin did not reduce *C. burnetii* replication.

## Materials and Methods 

### Reagents and Antibodies

Unless stated otherwise, reagents were purchased from Carl Roth, Sigma-Aldrich or Thermo Fisher. The following primary antibodies were used: anti-*Coxiella*, anti-AnkF (Davids Biotechnologie), anti-vimentin (Cell Signaling #5741S, or Sigma-Aldrich, #V6630), anti-tubulin (Cell Signaling, #3873) and anti-cytokeratin 18 (Thermo Fisher, #MA5-12104). Actin was stained with the Phalloidin-Alexa Fluor-647 conjugate (A2066, Sigma-Aldrich). The LAMP2 (ABL-93) specific primary antibody was developed by J.T. August and obtained from the Developmental Studies Hybridoma Bank, University of Iowa, Department of Biology, Iowa City, IA, USA. Secondary antibodies conjugated with Alexa Fluor-488 or -594 were purchased from Dianova. For STED confocal microscopy, Alexa Fluor-580-STAR antibodies (#2-0012-005-8, Abberior GmbH, Göttingen, Germany) were kindly provided by Dr. Ralph Palmisano, Optical Imaging Center Erlangen (OICE), Germany.

### Bacterial Strains, Yeast Strains, and Cell Lines

*Escherichia coli* DH10β were cultivated in Luria Bertani (1% bacto tryptone, 0.5% yeast extract and 1% NaCl) broth supplemented with 100 µg/ml ampicillin or 50 µg/ml kanamycin where appropriate. *Coxiella burnetii* Nine Mile II (NMII) RSA439 clone 4 were grown in acidified citrate-cysteine medium (ACCM-2) at 37°C, 2.5% O_2_, and 5% CO_2_. Axenic media were supplemented with 3 µg/ml chloramphenicol where appropriate for selection. The leucine- and tryptophan-auxotrophic *Saccharomyces cerivisiae* strains Y187, AH109, and Y190 were grown in YPAD (1% yeast extract, 2% caseine peptone, 2% glucose, and 0.01% adenine hemisulfate) or SCAD (2% glucose, 0.6% yeast nitrogen base, 0.06% amino acid mix, pH 5.8) with medium shaking or on agar plates (media supplemented with 1.5% agar) at 30°C.

CHO-FcR cells (Chinese hamster fibroblasts endogenously expressing the macrophage-lymphocyte Fc receptor) were maintained in Dulbecco's Modified Eagle´s Medium (DMEM, Thermo Fisher). HeLa (human cervical carcinomal epithelial cells), U2OS and U2OS-vimentin-rsEGFP (recombinant human bone osteosarcoma cells endogenously expressing vimentin-rsEGFP (Ratz et al., 2015)) were maintained in DMEM. HeLa cells stably transfected with pWHE644/655-AnkF were cultured in DMEM supplemented with 1% Penicillin/Streptomycin (Thermo Fisher), 0.3 mg/ml Geneticin (G418) and 0.25 μg/ml puromycin (Berens et al., 2015; Bisle et al., 2016). All media were supplemented with 5% heat-inactivated fetal bovine serum (FBS, Biochrom, Berlin, Germany) during infection with *C. burnetii* or 10% FCS when cells were cultured in the absence of bacteria.

### Analysis of *ankF* in 52 *C. burnetii* Strains

Genome assemblies of strains, which had been uploaded at the complete genome, chromosome, scaffold and contig levels and for which information on their genome group classification was known (Hemsley et al., 2019), were identified using the search term “*Coxiella burnetii*” in NCBI Genome (https://www.ncbi.nlm.nih.gov/genome/), downloaded and used to create a Coxiella-WGS database in Geneious Prime 2019.2.3 (Biomatters, New Zealand). Only a single sequence was taken from strains with multiple entries or passage variants. The respective *ankF* sequences were identified by BLAST analysis of Coxiella-WGS using the *ankF* coding sequence from the RSA493 Nine Mile strain as reference and the Geneious default parameters. Sequences from two strains were discarded due to sequence ambiguity (Cb171_QLYMPHOMA; CDBG01000000) and a partial sequence at the end of a contig (Q321; AAYJ01000000), so that 52 sequences remained for the mutational analysis.

### Analysis of *C. burnetii ankF*::Tn

*C. burnetii* and the transposon mutant *C. burnetii ankF*::Tn (*ankF::*Tn) were analyzed by PCR for the presence of *ankF* and the insertion of the transposon with the primers 608 and 609 amplifying the *ankF* codon sequence.

### Infection With *C. burnetii*

Infection of cell lines with *C. burnetii* was performed as described elsewhere (Schulze-Luehrmann et al., 2016). Briefly, *C. burnetii* were cultured axenically for 3 days at 37°C, 2.5 % O_2_ and 5 % CO_2_ and 1 day at RT and normal atmosphere. To infect cell lines, bacteria were pelleted and adjusted spectrophotometrically in PBS to yield respective MOIs. Bacteria were then added to 300 µl or 600 µl of cell culture media used for cells seeded 24 h earlier in 24-well or 6-well plates, respectively.

### Immunofluorescence

Cells seeded in 24-well plates were washed three times with 1 ml PBS and fixed with 4% paraformaldehyde (Alfa Aesar, Karlsruhe, Germany) in PBS for 15 min at RT. Following three wash steps with PBS, cells were permeabilized with 0.1% Triton X-100 (Sigma-Aldrich) in PBS for 3 min, washed again, and blocked and quenched with PBS containing 50 mM NH_4_Cl (Roth, Karlsruhe, Germany) and 5% Goat serum (Thermo Fisher) for 30 min at RT. Cells were subsequently incubated with primary antibodies in 5% Goat serum in PBS for 45 min at RT. Primary antibodies were washed off three times with PBS, and cells were incubated for 30 min at RT with fluorophore-coupled secondary antibodies in 5% Goat serum in PBS. Secondary antibodies were washed off three times with PBS, and stained cells were mounted on glass slides with ProLong Diamond containing DAPI to stain nuclei or bacterial DNA.

### Human Peripheral Blood-Derived Macrophages

From three healthy donors (Ethical Committee Erlangen approval number 111-12B) peripheral blood were obtained, and macrophages were derived as previously described (Hayek et al., 2019).

### Colony-Forming Units (CFU)

The infected primary macrophages were washed with PBS, incubated for 40 min in ice-cold H_2_O and pipetted repeatedly with a syringe carrying a 25G needle (25 G 1'' 0.5 mm × 25 mm, BD Microlance 3, Spain) to lyse the cells. The lysates were centrifuged (10 min, 1000 rpm, 4°C) and the supernatant was pelleted (1 min, 14000 rpm, 4°C) and resuspended in 200 µl PBS pH 7.4. A serial dilution was performed and pipetted in triplicates on ACCM-D/0.5% agarose plates. The plates were incubated for 2 weeks at 37°C, 5% CO_2_ and 2.5% O_2_ and the CFUs were counted.

### Phenotypic Screening

For phenotypic screening, samples were imaged with an ArrayScan VTI Live epifluorescence automated microscope (Cellomics) equipped with an ORCA-ER CCD camera (Hamamatsu). Twenty-five fields per well were acquired for image analysis. Phenotypic profiles (expressed as z-scores) were calculated using CellProfiler, from triplicate experiments as previously described (Martinez et al., 2015) following median based normalization of 96-well plates. Plate effects were corrected by the median value across wells that are annotated as control.

### Labeling of DQ-Red BSA–Positive Proteolytic Organelles

HeLa cells were infected with *C. burnetii* or the transposon mutant *ankF*::Tn at an MOI of 50. Following infection, cells were incubated with 10 µg/ml DQ-Red BSA (Life technologies) for 16 h. Cells were subjected to immunofluorescence staining as described.

### Generation of the *C. burnetii* Δ*ankF* Mutant and the Complemented Strain

*C. burnetii* Nine Mile phase II was electroporated with 10 µg pJC-CAT::*ankF*-5´3´-*lysCA* as previously described (Beare and Heinzen, 2014). Co-integrants were selected by culturing the bacteria in ACCM-D media lacking lysine, but containing 2% sucrose for 5 days as previously described (Beare et al., 2018). Surviving transformants were expanded in ACCM-D media lacking lysine for 7 days. After spreading the diluted culture on 0.25% ACCM-D agarose without lysine, clonal Δ*ankF* mutants were picked after 10 days of culture. The picked clones were expanded in ACCM-D media without lysine.

Complementation of Δ*ankF* was achieved by electroporation of the mutant strain with 10 µg pMiniTn7T-*ankF*::AnkF. Integrants were selected by culturing the bacteria in ACCM-D media lacking lysine and lacking arginine, but containing citrulline for 5 days as previously described (Beare et al., 2018). The diluted culture was spread on 0.25% ACCM-D agarose without lysine and arginine, but containing citrulline for 10 days. Individual clones were picked and expanded in ACCM-D medium lacking lysine and arginine, but with citrulline.

### Preparation of a HeLa cDNA Library for the Yeast Two-Hybrid Assay 

A cDNA library (Clontech, #HL4000AA) from the HeLa S3 cell line was kindly provided by Prof. Dr. Hashemolhosseini (Institute for Biochemistry, University of Erlangen-Nuremberg). The cDNA library was restricted with EcoRI and XhoI and ligated into the de-phosphorylated and likewise-digested vector pGAD-GH.

### Transformation of *S. cerevisiae* Y187 With Plasmid DNA

To transform the tryptophan- and leucine-auxotrophic *S. cerevisiae* Y187 with pGBT9-AnkF (prey-construct), a single yeast colony was grown on complete YPAD agar at 30°C. The next day, a colony was used for inoculation of YPAD medium at 30°C and 170 rpm shaking. The culture was grown to mid-logarithmic growth phase and sequentially washed in sterile ddH_2_O and twice in Lithium-acetate (LiAc). After another centrifugation step the pellet was re-suspended in 50% PEG 3350, 1 M LiAc, 5 μl of 10 mg/ml salmon sperm DNA (Invitrogen) and 3 µg of pGBT9-AnkF plasmid DNA. The transformation was performed via the heat-shock method at 42°C for 20 min. Next, the culture was pelleted, re-suspended in ddH_2_O, plated dropwise onto SCAD agar plates lacking tryptophan (SCAD^-T^) for selection and incubated 2 to 3 days at 30°C.

### Transformation of *S. cerevisiae* AH109 With a HeLa Genomic Library

*S. cerevisiae* AH109 were grown as described above. The next day, the mid-log phase culture was washed sequentially in sterile ddH_2_O and TE/LiAc-buffer (10 mM Tris pH 7.4, 1 mM EDTA, 100 mM LiAc) including 10 mg salmon sperm DNA and 3 mg of purified HeLa genomic Library. The yeast suspension was subsequently incubated for 30 min at 30°C and another 15 min at 42°C supplemented with 10% dimethyl sulfoxide (Sigma-Aldrich). Following a 2-min incubation on ice, the suspension was re-suspended in ddH_2_O, plated on SCAD plates lacking tryptophan (SCAD^-T^) and incubated for 3 days at 30 °C. The colonies were pooled in YPAD medium with 20% w/v glycerol and further cultured at 30°C for 2.5 h. Respective cultures were aliquoted and stored at −80°C.

### Yeast Two-Hybrid Screen

The Yeast Two Hybrid screen was performed by mating the yeast strain *S. cerevisiae* Y187-pGBT9-AnkF with the strain *S. cerevisiae* AH109 containing the HeLa genomic library. For this purpose, a pre-culture of *S. cerevisiae* Y187-pGBT9-AnkF was inoculated and cultivated in SCAD^-T^-medium at 30°C and 170 rpm shaking. Next, a SCAD^-T^ over-night (ON) culture was inoculated and incubated at 30°C and 170 rpm shaking until reaching an OD_600_ of approximately 0.8 to 1.2. Cultures were pelleted at 4,500*g* at RT. Meanwhile, two aliquots of the *S. cerevisiae* AH109 strain containing the HeLa genomic library were thawed in a 30°C water bath and subsequently added to the pelleted *S. cerevisiae* Y187-pGBT9-AnkF culture. The culture mix was vortexed, pelleted again, re-suspended in residual supernatant and subsequently plated onto YPAD plates. Following a 4.5-h incubation at 30°C, clones were washed off the YPAD plates twice with medium and pelleted. Next, the pellet was re-suspended in water, plated onto selective SCAD plates lacking histidine, leucine, and tryptophan (SCAD^-HLT^) and incubated for five days at 30°C for clonal isolation.

### *LacZ* Filter-Lift Assay

Clones of the mated yeast strains *S. cerevisiae* Y187-pGBT9-AnkF and *S. cerevisiae* AH109 grown on selective SCAD ^–HLT^ plates were considered to harbor both bait- and prey plasmids and to express the GAL4 transcription factor and the HIS3 gene. GAL4 initiates expression of the *lacZ* reporter gene serving as a read out for bait (AnkF) and prey (HeLa genomic library) interaction. HIS3 serves to complement the histidine auxotrophy.

Mated yeasts were plated onto nitrocellulose membranes placed on SCAD^-HLT^ plates and incubated at 30°C for 3 days. To read out the LacZ reporter activity, nitrocellulose membranes were drowned in liquid nitrogen and then placed onto filter paper soaked in 2 ml LacZ buffer (60 mM Na_2_HPO_4_, 40 mM NaH_2_PO_4_, 10 mM KCl, 1 mM MgSO_4_, pH 7.0), supplemented with 66.2 µl 2 % X-Gal (Biomol) in dimethylformamide and 5.4 µl beta-mercaptoethanol). Following another incubation step of 3 to 5 h at 30°C, respective clones were checked for blue dye precipitation on nitrocellulose membranes.

### Reverse Transcription-Quantitative Real-Time PCR (RT-qPCR) of Vimentin in Presence of AnkF

For isolation of RNA, 1.2 × 10^6^ stably-transfected HeLa-pWHE644/655-AnkF cells were seeded in 6-well plates in 3 ml per well and induced for AnkF expression with 1 μg/ml doxycycline. Cells were washed with PBS and lysed with RLT buffer (RNeasy® Plus Mini Kit, Qiagen, Germany) supplemented with β-mercaptoethanol. Total RNA was isolated using the RNeasy® Plus Mini Kit (Qiagen) and the QIAshredder™ kit according to manufacturer's instructions. RNA was eluted from columns in RNAse-free ddH_2_O. The reverse transcription of RNA into cDNA was performed with the SuperScript™ II Reverse Transkriptase kit (Thermo Fisher) with random Oligo(dT)_12-18_ primers (Thermo Fisher). QPCR was performed to quantify the amount of vimentin cDNA using primers 936 and 937 and quantification of actin cDNA using primers 827 and 828 (**Table 1**) with the QuantiFast SYBR Green PCR kit (Qiagen, Germany). Amplification of cDNA was performed in 384-well optical plates in an ABI Prism 7900HT. Relative amounts of vimentin cDNA were calculated by the ΔΔCT method using the housekeeping gene GAPDH for normalization.

**Table 1:** Primers used in this study.

No.	Sequence (5′->3′)*	Restriction site
40	AAGGATCCCTACCGCTGGAAGCCGC	BamHI
53	CCGGATCCATGAGACAGCGTGAAATTAATG	PstI
79	CCGGTACCCTACCGCTGGAAGCCGC	KpnI
608	ATGCGCCAGCGTGAAATTAATGATGAAGCTAT	
609	CTACCGCTGGAAGCCGCGATTATTGTGTTTTT	
650	CCGGATCCTTAGACAGCGTGAAATTAATGAT	BamHI
672	AATTTCATCGTTCCCGGCAG	
673	GCCGCGTTTACTAATCCCCA	
710	GCGAATTCATATGTCCACCAGGTCCGTGT	EcoRI
711	GCCGGTACCTTATTCAAGGTCATCGTGATGC	KpnI
827	CCAACCGCGAGAAGATGA	
828	CCAGAGGCGTACAGGGAT	
936	TCCAGCAGCTTCCTGTAGGT	
937	CCC TCACCTGTGAAGTGGAT	
1063	CAGGAAACAGAATTCATGGTGTCAAAAGGAGAAGAAG	EcoRI
1065	AGAGGTACCGAGCTCTTATTTATAAAGTTCATCCATGCC	SacI

*Restriction sites are underlined.

### Immunoblot Analysis

Proteins were separated by SDS-PAGE Bolt™ Bis-Tris Plus gradient 4% to 12% polyacrylamide gels (Thermo Fisher) and transferred to 0.45 µm pore size Immobilon-P PVDF membranes (Merck Millipore) by the semi-dry transfer method. Blotted membranes were incubated with respective primary antibodies and HRP-conjugated secondary antibodies. Immunodetection was performed with the Pierce ECL Western Blotting Substrate (Thermo Fisher) according to the manufacturer's instructions and exposure of X-ray films (GE Healthcare) to chemiluminescence.

### Transient Transfection of CHO or HeLa Cells

2 × 10^4^ *C. burnetii*-infected or uninfected cells were seeded on coverslips in a 24-well plate in 1 ml medium and incubated at 37°C, 5% CO_2_ for 24 h. Cells were transfected with 250 to 500 ng of plasmid DNA using the X-tremeGENE 9 DNA Transfection Reagent (Roche, Switzerland) following the manufacturer´s protocol.

### Confocal Microscopy

Fixed and stained cells on cover slips were mounted on glass slides and visualized with a Zeiss LSM 700 confocal laser scanning microscope. Image acquisition was performed with Zeiss Zen software (Carl Zeiss, Oberkochen, Germany).

### STED Microscopy

For high-resolution STED microscopy, fixed and stained cells on cover slips (12 mm radial cover slips, 0.17± 0.005 mm) were visualized with use of an Abberior 3D STED 2-Channel Super Resolution- and resolft microscope (Abberior Instruments GmbH, Göttingen, Germany). Images were acquired with the Imspector software (Abberior GmbH, Göttingen, Germany).

### Live Cell Imaging

U2-OS-vimentin-rsEGFP cells infected with *C. burnetii*-tdTomato were cultivated in µ-slides (Ibidi, Planegg, Germany) and visualized with a Zeiss Spinning Disc Axio Observer Z1 (Carl Zeiss, Oberkochen, Germany).

### siRNA-Mediated Knock-Down of Vimentin in HeLa Cells and Quantification of Intracellular *C. burnetii*

Transfection of 1 × 10^5^ HeLa cells in 12-well plates was performed by transfection of a 50 nM On-Targetplus human siRNA pool (Dharmafect) specific for human vimentin (vim) or a non-targeting siRNA pool as a control with the DharmaFECT 1 transfection reagent (Thermo Fisher) according to the manufacturer's instructions. Following a 24-h incubation at 37°C and 5% CO_2_, cells were infected with *C. burnetii*. At the indicated time-points post-infection, *C. burnetii* was isolated from HeLa cells by osmotic lysis in sterile ddH_2_O. In detail, *C. burnetii* infected HeLa cells were washed and subsequently lysed with 2 ml sterile ddH_2_O for 30 min at 37°C and 5% CO_2_. Lysed cells were re-suspended thoroughly, and bacteria were isolated by differential centrifugation at 300*g* for 10 min at RT and afterward at 20.000*g* for 2 min at RT. For isolation of *C. burnetii* genomic DNA (gDNA), pelleted *C. burnetii* were processed using the Illustra Bacteria Genomic Prep Mini Spin Kit (GE Healthcare) according to the manufacturer's instructions. Isolated gDNA from axenically-grown *C. burnetii* was used as a genomic equivalent (GE) standard ranging between 10^3^ and 10^7^ copies for absolute quantification. Calculation of GEs for the standard was performed as described elsewhere (Schulze-Luehrmann et al., 2016). Amplification of the *IS1111* insertion sequence was performed in 384-well optical plates in an ABI Prism 7900HT using primers 672 and 673 (**Table 1**).

### Plasmid Construction

Restriction enzymes were purchased from NEB or Thermo Scientific. The antarctic phosphatase and T4-ligase were purchased from Thermo Fisher. Primer sequences and constructed plasmids are listed in **Table 2** and **Table 3**, respectively. For creation of pCMV-HA-vimentin, the vimentin coding sequence was amplified for cloning from isolated HeLa genomic DNA with the primers 710 and 711 by PCR with Q5-Phusion polymerase (NEB), purified, restricted with EcoRI and KpnI and ligated into the likewise-restricted and de-phosphorylated vector pCMV-HA. To create pEGFP-C2-AnkF, the AnkF coding sequence was amplified from *C. burnetii* NMII genomic DNA with primers 53 and 79, restricted with PstI and KpnI and ligated into the likewise-restricted and de-phosphorylated vector pEGFP-C2. For construction of pKM244mod.-tdT_cc_, a *Coxiella*-codon optimized coding sequence of tandem-di-tomato (tdT_cc_), synthesized and cloned into pEX-K4 (pEX-K4-tdT_cc_), was ordered from Eurofins (Luxemburg). The tdT_cc_- coding sequence was amplified by PCR from pEX-K4-tdT_cc_ with the primers 1063 and 1065 and cloned into the EcoRI-digested vector pKM244mod with use of the GeneArt® Seamless Cloning and Assembly Kit (Thermo Fisher) according to the manufacturer's instructions to create pKM244mod.-tdT_cc_. The plasmid pGBT9-AnkF was cloned by PCR from the AnkF coding sequence of *C. burnetii* NMII genomic DNA with the primers 650 and 40 followed by fragment purification, restriction with BamHI and ligation into the likewise-restricted and de-phosphorylated vector pGBT9.

**Table 2:** Plasmids constructed in this study.

Plasmid	Primers	Reference
pCMV-HA-vimentin	No. 53 and No. 79	This study
pEGFP-C2-AnkF	No. 710 and No. 711	This study
pKM244mod.-tdT_cc_	No. 1063 and No. 1065	This study
pJC-CAT::*ankF*-5′3′-*lysCA*		This study
pMini-Tn7T-*ankF*::AnkF		This study

**Table 3:** Analysis and grouping of the *ankF* gene in 52 *C. burnetii* strains.

Genome Group	Strain	AnkF group	Accession #	Sequence Reference
**I**	RSA493 (NM-I)	1	AE016828	(Seshadri et al., 2003)
	RSA315 (Turkey)	1	NOLO00000000	(Beare et al., 2017b)
	RSA435 (Dyer)	1	NOLQ00000000	(Beare et al., 2017b)
	RSA270 (Ohio314)	1	NOLT00000000	(Beare et al., 2017c)
	RSA329 (California33)	1	NOLV00000000	(Beare et al., 2017c)
	RSA350 (California16)	1	NOLU00000000	(Beare et al., 2017c)
	RSA514 (NM-Crazy)	1	NOVG00000000	(Beare et al., 2018)
	Cb_C2	1	CCAJ010000000	(Sidi-Boumedine et al., 2014)
**(I)**	Cb175_Guyana	1	HG825990	(D’Amato et al., 2015)
**IIa**	RSA331 (Henzerling)	1	CP000890, CP014559	(Beare et al., 2006; Kuley et al., 2017)
	Heizberg	1	CP014561	(Kuley et al., 2017)
	RSA461 (M44_Clone1)	1	NOVI00000000	(Beare et al., 2018)
	Cb185	1	CBTH01000000	(Million et al., 2014)
**IIb**	CbCVIC1	1	CP014549	(Kuley et al., 2017)
	Z3055	1	LK937696	(D’Amato et al., 2014b)
	NL-Limburg	1	JZWL01	(Hammerl et al., 2015)
	NL3262	1	CP013667	(Kuley et al., 2016)
	NLhu3345937	1	CP014354	(Kuley et al., 2016)
	602 (14160-002)	1	CP014836	(Kuley et al., 2017)
	42785537	1	CP014548	(Kuley et al., 2017)
	EV-Cb_C13	1	CCAM010000000	(Sidi-Boumedine et al., 2014)
	Q540	1	PPFP01000000	(Hemsley et al., 2019)
	Cb_D2 (DSTL_2)	1	RQJT01000000	(Hemsley et al., 2019)
	Cb_D8 (DSTL_8)	1	RQJS01000000	(Hemsley et al., 2019)
	Cb_D10 (DSTL_10)	1	RQJR01000000	(Hemsley et al., 2019)
	Cb109	1	AKYP01000000	(Rouli et al., 2012)
**III**	Idaho Goat_Q195	1	NOLR00000000	(Beare et al., 2017c)
	2574	1	CP014555	(Kuley et al., 2017)
	601 (14160-001)	1	CP014551	(Kuley et al., 2017)
	18430	1	CP014557	(Kuley et al., 2017)
	701CbB1	1	CP014553	(Kuley et al., 2017)
	Cb_B1	1	CCAH010000000	(Sidi-Boumedine et al., 2014)
	Cb_B18	1	CCAI010000000	(Sidi-Boumedine et al., 2014)
	EV-Cb_BK18	1	CCAL010000000	(Sidi-Boumedine et al., 2014)
	Q532	1	PPFQ01000000	(Hemsley et al., 2019)
	Q545	1	PPFO01000000	(Hemsley et al., 2019)
	Cb_D1 (DSTL_1R)	1	RQJU01000000	(Hemsley et al., 2019)
	Q556	1	PPFN01000000	(Hemsley et al., 2019)
	Q559	1	PPFM01000000	(Hemsley et al., 2019)
**IV**	Schperling	4	CP014563	(Kuley et al., 2017)
	Cbu_K154	4	CP001020	(Beare et al., 2009)
	‘MSU Goat Q177 (Priscilla)	4	CP018150	(Walter et al., 2016) Unpublished
	Leningrad-2	4	PDLP00000000	(Freylikhman et al., 2017) Unpublished
	Namibia	5	CP007555	(Walter et al., 2014)
	AuQ01 (Arandale)	4	JPVV01000000	(Walter et al., 2014)
	Cb196_SaudiArabia	4	CCXO01000000	(D’Amato et al., 2014b)
	Cb_O184	4	CCAK010000000	(Sidi-Boumedine et al., 2014)
**V**	Cbu G_Q212	2	CP001019	(Beare et al., 2009)
	Scurry S_Q217	2	CP014565	(Kuley et al., 2017)
	Ko_Q229	2	NOLP00000000	(Beare et al., 2017b)
	Dog Utad	2	CCNR01000000; CCYB01000000	(D’Amato et al., 2014a)
**VI**	Dugway 5J108-111, 7D77-80, 7E65-68	3	CP000733; NOLN01000000, NOLM01000000	(Beare et al., 2009; Beare et al., 2017a)

For construction of pJC-CAT::*ankF*-5′3′-*lysCA*, the 5′ and 3′ regions of *ankF* were amplified by PCR from NMII genomic DNA using the specific primer sets (5′-CGGTACCCGGGGAT CCCATATCGATAATGTGTTGATGG and 3′-CACCCATATGCGACGCGAGCGTCGA GTTCTTTCTCTACCTAATTAAACTTTATG) and (5′-CGTCGCATATGGGTGCGCATG TACGTCTCCGCTAAGTAGCCCGTATG and 3′-GAACCTGTTTGTCGACGCTTGAGA TTCAGCGGGTGG), respectively. The 5′ and 3′ PCR products were cloned into BamHI/SalI-digested pJC-CAT by In-Fusion, resulting in formation of an internal NdeI site between the 5′ and 3′ fragments and creation of pJC-CAT::*ankF*-5′3′. The *1169^P^-lysCA* cassette was amplified from pJC-CAT::*1169^P^-lysCA* (Beare et al., 2018) by PCR with specific primers a450 and a451 and cloned by In-Fusion into NdeI-digested pJC-CAT::*ankF*-5′3′ to create pJC-CAT::*ankF*-5′3′*-lysCA*.

For construction of pMini-Tn7T-*ankF*::AnkF the *ankF* gene and its native promotor were amplified by PCR from NMII genomic DNA using the specific primers (5´-GATGAATTCGACGAGCAAAGGAGCCCT and 3´-GTAGAATTCTTCGCCATCTTC TTAGCGCAC) followed by fragment purification, restriction with EcoRI and ligation into the likewise-digested and de-phosphorylated vector pMini-Tn7T-ArgGH (Sandoz et al., 2016; Beare et al., 2018).

### Statistical Analysis

Statistical analysis was conducted with Prism 8 (GraphPad software). Bar graphs depict mean data ± standard deviation from three independent experiments. An unpaired Student's t-test was performed to determine significance of each data point. A *p*-value of < 0.05 was considered significant.

## Results

### AnkF Is a Highly Conserved Effector Protein

**Figure 1:**
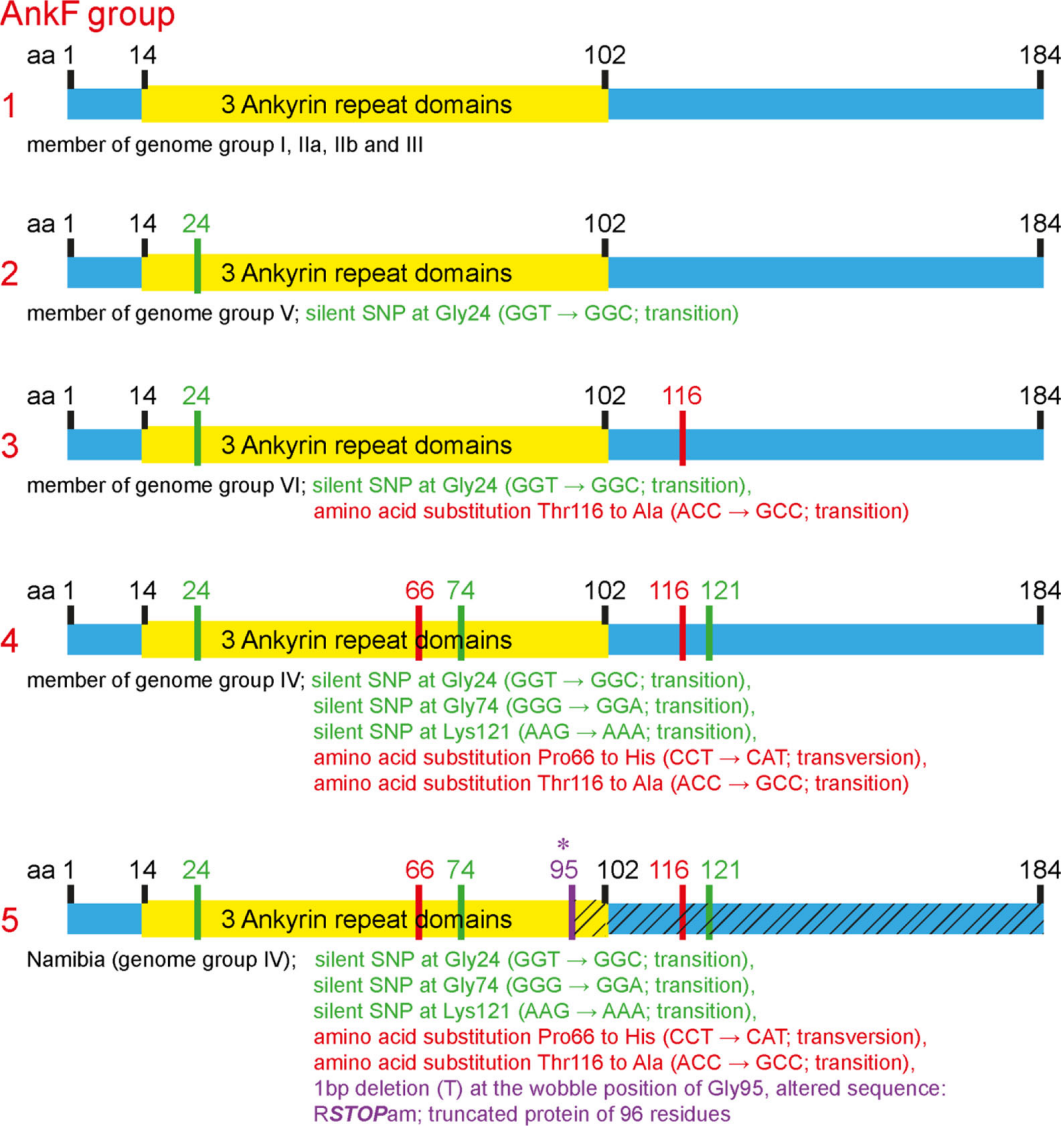
Schematic diagram of five alleles of the *ankF* gene. The sequences of the respective *ankF* gene from 52 *C. burnetii* strains (**Table 3**) were analyzed. Depicted are the three ankyrin repeat domains (in yellow), silent nucleotide polymorphisms (in green), nucleotide exchanges (in red) and a frameshift mutation leading to a premature stop (in purple; marked with *). Beneath the diagram of the alleles, the corresponding *C. burnetii* genome group and the nucleotide substitutions are given.

The T4SS effector protein AnkF was one of the first *C. burnetii* T4SS effector proteins identified (Pan et al., 2008). However, its function has not been studied in detail so far. In a previous publication, the *ankF* sequences from four different *C. burnetii* isolates were analyzed. As the *ankF* gene appeared to be highly conserved (Voth et al., 2009), it was suggested that AnkF might be an important virulence factor. In the meantime, additional *C. burnetii* isolates have been sequenced. Thus, we compared the *ankF* sequences from 52 *C. burnetii* strains (**Table 3**). Our analysis demonstrates that these strains encode five different alleles of the *ankF* gene (**Figure 1**). Isolates assigned to the genome groups I, IIa, IIb, and III (Hemsley et al., 2019; Long et al., 2019) express the wild-type sequence of the Nine Mile reference strain. All genome group V members contain a silent single-nucleotide polymorphism (SNP) at Gly24, thus, also encode a wild-type protein. The Dugway strain, representing genome group VI, encodes a full-length protein with the Gly24 SNP and an additional amino acid substitution from threonine to alanine at residue 116. The *ankF* genes from strains classified as belonging to genome group IV contain the latter two mutations. In addition, they also contain silent SNPs in the codons for Gly74 and Lys121 as well as a mutation leading to a proline to histidine exchange at position 66. There is only one exception to this sequence/genome group correlation in genome group IV. Here, the Namibia strain has an additional frameshift mutation at residue Gly95, resulting in a protein of 96 residues. Overall, our analysis suggests that *ankF* is highly conserved between the different isolates, which supports the assumption that AnkF might be an essential virulence factor.

### AnkF Transposon Mutants Are Infectious but Fail to Establish a Replicative CCV

**Figure 2:**
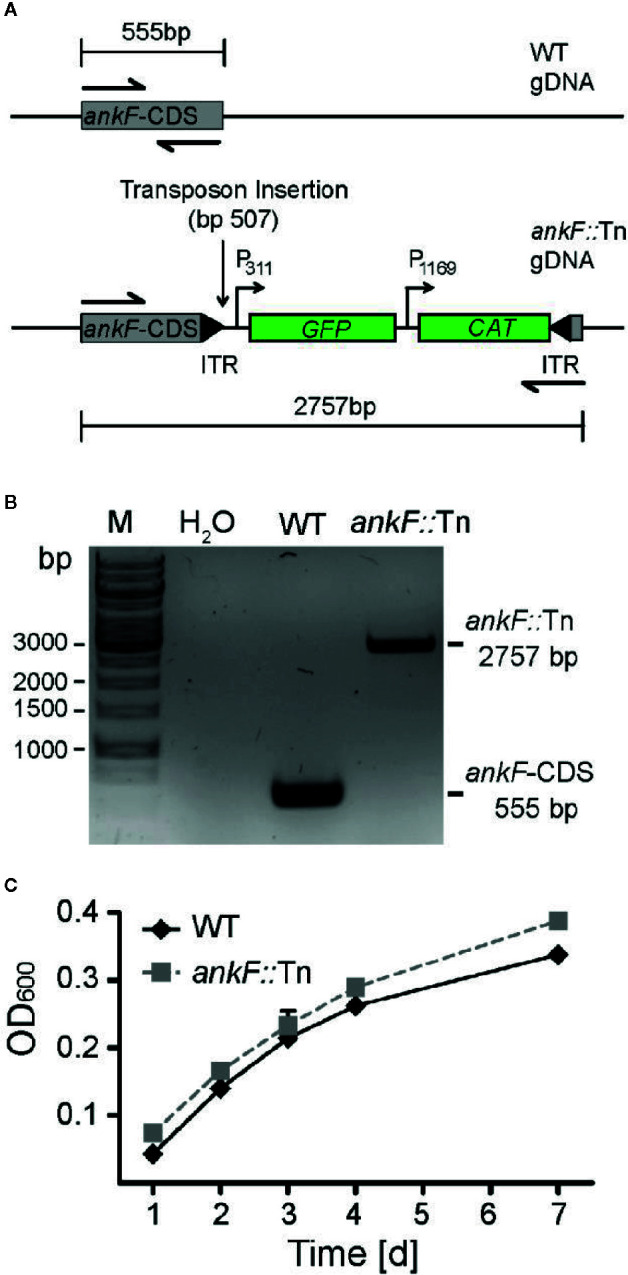
The *ankF*::Tn mutant has no replication defect in axenic culture. **(A)** Scheme of the genomic region of wild-type *C. burnetii* and *C. burnetii*-*ankF*::Tn at the *ankF* locus. The transposable element is inserted between bp 507 and 508 of the *ankF* coding sequence and contains a GFP reporter under the control of the *C. burnetii* promotor *P_311_* and a chloramphenicol resistance cassette under the control of the *C. burnetii* promotor *P_1169_*. **(B)** Agarose gel of PCR products from wild-type *C. burnetii* and *ankF*::Tn generated with primers specific for the *ankF* coding sequence (shown as halved arrows in A). **(C)** Wild-type *C. burnetii* and *ankF*::Tn were inoculated at an OD_600_ of 0.01 in ACCM-2 medium and incubated at 37°C, 2.5% O_2_ and 5% CO_2_. OD_600_ was determined by spectrophotometric analysis at the indicated time-points post-inoculation. Error bars represent the mean standard deviation of three independent experiments.

**Figure 3:**
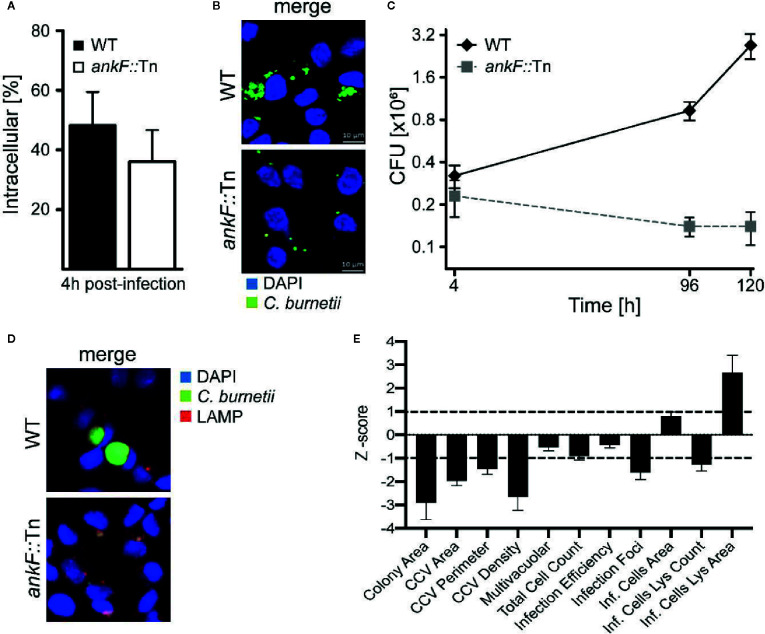
The *C. burnetii* T4SS effector AnkF is essential for intracellular replication. **(A, B)** HeLa cells were infected with wild-type *C. burnetii* (WT) and *ankF*::Tn (*ankF*::Tn) at an MOI of 50. **(A)** 4 h post-infection the number of intracellular bacteria was determined from 100 infected cells. Error bars represent mean standard deviations of three independent experiments. **(B)** 48 h post-infection, the cells were fixed and stained with antisera against *C. burnetii* (green). Nuclei and bacterial DNA were stained with DAPI (blue). Representative LSM images are shown. **(C)** Human monocyte-derived macrophages were infected with wild-type *C. burnetii* (WT) and *ankF*::Tn (*ankF*::Tn) at an MOI of 10 for 4, 96, and 120 h. The bacterial numbers were determined by colony-forming unit (CFU) counts. Error bars represent the mean standard deviation of three independent experiments. **(D, E)** U2OS cells were challenged either with wild-type *C. burnetii* or the *ankF*::Tn mutant strain, both expressing GFP, at an MOI of 100. Six days post-infection, cells were fixed and labeled with an anti-LAMP1 antibody (red) and Hoechst (blue) to visualize CCVs and host cell nuclei, respectively. **(D)** Images were acquired with an Arrayscan VTI Live epifluorescence automated microscope equipped with a 20× objective and an ORCA ER CCD camera. Representative images are shown. **(E)** An average of 50,000 cells were then automatically imaged and analyzed from triplicate experiments for each condition and the phenotypic profile of the *ankF*::Tn mutant was compared to that of wild-type *C. burnetii* and expressed as z-scores over 11 independent features.

In order to determine whether AnkF is involved in *C. burnetii* pathogenesis, we analyzed AnkF mutants in their ability to infect cells and to replicate intracellularly. To this end we used an *ankF* transposon mutant of *C. burnetii* (*ankF*::Tn), which harbors a transposable element integrated between bps 507 and 508 of the *ankF* coding sequence (**Figure 2A**). First, we confirmed clonality by performing a PCR of wild-type and *ankF*::Tn *C. burnetii* (**Figure 2B**). Next, we analyzed the capability of the transposon mutant to grow in axenic culture. As shown in **Figure 2C**, axenic growth of the mutant is comparable to that of the wild-type strain over seven days. In order to elucidate the role of AnkF during infection, the transposon mutant was characterized regarding internalization and intracellular replication. Thus, HeLa cells were infected with the *ankF* mutant and wild-type *C. burnetii* and immunofluorescence was performed at 4 and 48 h post-infection. At 4 h post-infection, roughly 40% to 50% of the cells contained intracellular bacteria for each condition, implying that the *ankF*::Tn and wild-type strains are equally infectious (**Figure 3A**). However, *ankF*::Tn failed to replicate intracellularly in HeLa cells (**Figure 3B**), primary human monocyte-derived macrophages (**Figure 3C**), and in U2OS cells (**Figure 3D**), demonstrating that this replication defect is not cell-type specific. Next, to further investigate the replication defect of *ankF*::Tn mutants, we performed multi-parametric phenotypic profiling. For this purpose, we resorted to U2OS cells, as their morphology simplifies phenotypic characterization. In addition to their replication defect within infected cells, the *AnkF*::Tn mutants were impacted in their ability to develop CCVs (**Figure 3E**). Accordingly, CCVs formed by the *ankF*::Tn mutants harbored less bacteria as compared to wild-type. Other parameters were largely unaffected, with the exception of the area occupied by lysosomes in infected cells, which is consistent with a defect in CCV biogenesis. Thus, the *ankF*::Tn mutant has a defect in intracellular replication, which might be caused by disturbed CCV development.

### AnkF Is Dispensable for CCV Maturation, but Might Act on CCV Characteristics

**Figure 4:**
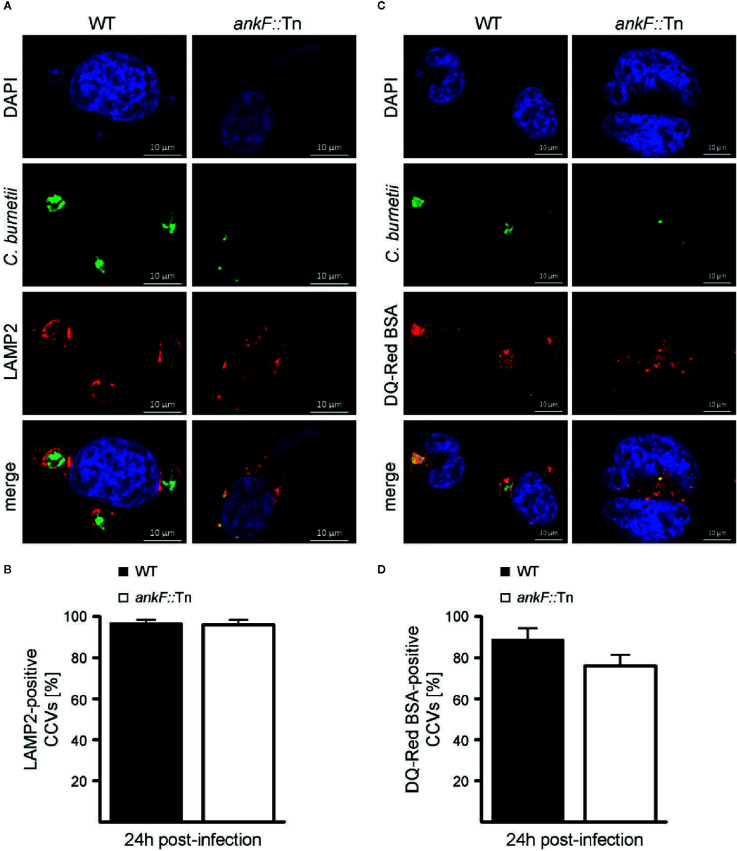
The *C. burnetii* T4SS effector AnkF is dispensable for CCV maturation. **(A, B)** HeLa cells were infected with wild-type *C. burnetii* (WT) and the *ankF*::Tn (*ankF*::Tn) mutant at an MOI of 50. At 24 h post-infection the cells were fixed and stained with an anti-LAMP2 antibody (red) and with antisera against *C. burnetii* (green). Nuclei and bacterial DNA were stained with DAPI (blue). Cells were visualized using LSM. **(A)** Representative LSM images are shown. **(B)** 100 infected cells were analyzed for the association of LAMP2 with the CCV. Error bars represent mean standard deviations of three independent experiments. **(C, D)** HeLa cells were infected with wild-type *C. burnetii* (WT) and *ankF*::Tn (*ankF*::Tn) at an MOI of 50. Cells were incubated with DQ-Red BSA for 16 h. At 24 h post-infection, the cells were fixed and stained with antisera against *C. burnetii* (green). Nuclei and bacterial DNA was stained with DAPI (blue). Cleaved DQ-Red BSA emits fluorescence (red). Cells were visualized using LSM. **(C)** Representative LSM images are shown. **(D)** 100 CCVs were analyzed for red fluorescence using an epifluorescence microscope. Error bars represent mean standard deviation of three independent experiments.

*C. burnetii* requires the maturation of the CCV into a lysosomal-like compartment with an acidic pH (van Schaik et al., 2013; Lührmann et al., 2017; Newton et al., 2020). Thus, immunofluorescence was performed with infected HeLa cells to monitor the presence of the lysosomal marker LAMP2 on the CCV. Additionally, the degradative activity of the CCV lumen was visualized by fluorescent fluorophore-coupled BSA (DQ-Red BSA) using LSM. DQ-Red is a self-quenched substrate that emits fluorescence after cleavage by proteases. The presence of LAMP2 around the CCV and fluorescent DQ-Red BSA inside the CCV was prominent at 24 h post-infection in HeLa cells infected with wild-type *C. burnetii* (**Figures 4A–D**). Importantly, the small CCVs formed by *ankF*::Tn were also decorated with LAMP2 and possessed a degradative lumen (**Figures 4A–D**). These data suggest that the inability of *ankF*::Tn to replicate intracellularly was not mediated by altered maturation of the CCV.

### The Replication Defect of AnkF Mutants Can Be Partially Complemented

**Figure 5:**
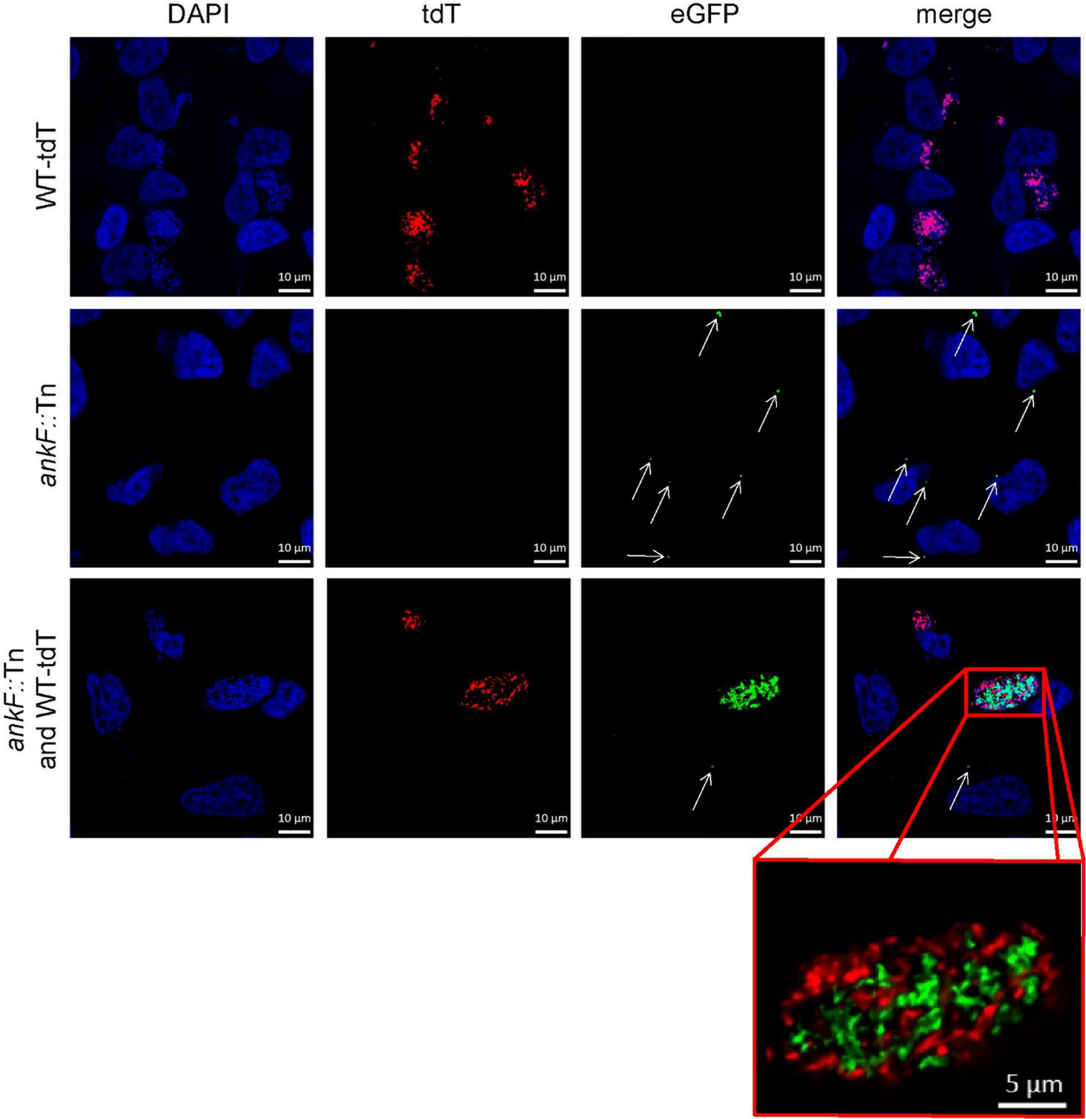
The AnkF transposon mutant replicates inside a CCV harboring wild-type bacteria. HeLa cells were infected with *C. burnetii* expressing IPTG-inducible tdTomato (WT-tdT) in the presence of 2 mM IPTG and/or *ankF*::Tn (*ankF*::Tn) endogenously expressing GFP at an MOI of 50 each as indicated. At 72 h post-infection, the cells were fixed and nuclei and bacterial DNA was stained with DAPI (blue). Representative LSM images of two independent experiments are shown. A red box represents a close-up without DAPI staining of the merged image. White arrows indicate single *ankF*::Tn cells.

Confirmation of loss-of-function studies by transposon mutagenesis requires phenotypic complementation. For this purpose, we infected HeLa cells with an equal MOI of both tdTomato-expressing wild-type *C. burnetii* and *ankF*::Tn mutants expressing GFP and analyzed CCV formation and bacterial replication by immunofluorescence. In cells infected with only a single bacterium we observed at 72 h post-infection either a spacious CCV in case of wild-type bacteria or small CCVs in case of the transposon mutant (**Figure 5**). However, if wild-type and *ankF*::Tn bacteria share the same vacuole, we observed high numbers of wild-type and *ankF*::Tn bacteria (**Figure 5**). Importantly, we never detected such high numbers of *ankF*::Tn bacteria if the wild-type *C. burnetii* were in a different CCV, but still in the same cell. Thus, we reasoned that AnkF might be altering the CCV in a way, which allows bacterial replication.

**Figure 6:**
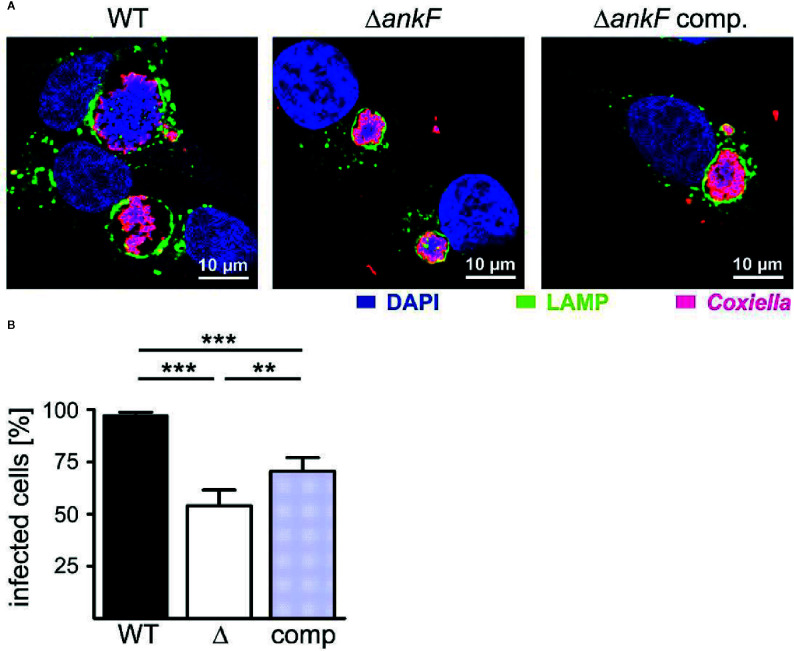
The replication defect of Δ*ankF C. burnetii* can be complemented. HeLa cells were infected with *C. burnetii* wild-type (WT), Δ*ankF* or Δ*ankF*::AnkF at an MOI of 100. At 60 h post-infection, the cells were fixed and stained with an anti-LAMP2 antibody (green) and with antisera against *C. burnetii* (red). Nuclei and bacterial DNA were stained with DAPI (blue). **(A)** Representative LSM images of three independent experiments are shown. **(B)** The percentage of infected cells were determined by analyzing 100 cells per experiment in three independent experiments. Error bars indicate ± SD. **p < 0.01, ***p < 0.001.

Next, we generated a clean AnkF deletion mutant (Δ*ankF*) and the respective complemented strain to prove the importance of AnkF for intracellular replication. Thus, we infected HeLa cells with wild-type, Δ*ankF* and Δ*ankF*::AnkF and analyzed the size of the CCVs and the infection rates at 14 and 60 h post-infection by immunofluorescence microscopy. As shown in **Figure 6A** at 60 h post-infection, wild-type *C. burnetii* establishes large CCVs, while the size of the CCVs harboring *C. burnetii* lacking AnkF were significantly smaller and the CCVs containing the complemented strain showed an intermediate vacuole size. In addition, while the infection rate at 14 h post-infection was 80% to 90% and did not differ between the three different strains (data not shown), at 60 h post-infection the infection rate of cells infected with the AnkF deletion mutant were reduced by ~40% in comparison to cells infected with wild-type *C. burnetii*. The complemented strains showed an intermediate phenotype (**Figure 6B**). These data supports the assumption that the T4SS effector protein AnkF is involved in intracellular replication of *C. burnetii*.

Taken together, our results demonstrate that AnkF is also important for *C. burnetii* replication in macrophages and, thus, in the primary host cell of *C. burnetii*. In addition, our results suggest, that AnkF might influence CCV properties.

### AnkF Binds Vimentin and Alters Its Localization

**Figure 7:**
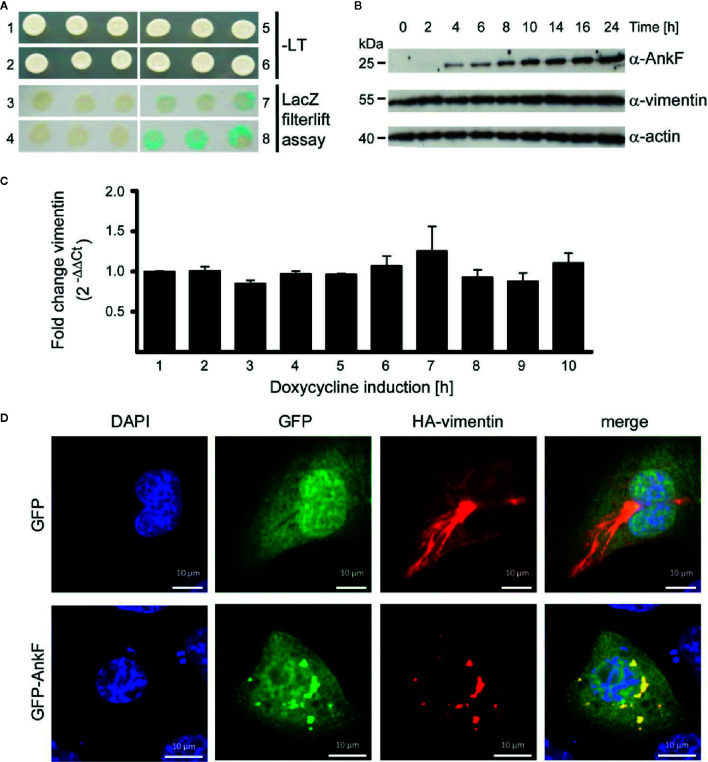
AnkF binds the type III intermediate filament vimentin. **(A)** A yeast-two-hybrid assay was performed with the Matchmaker Gold Yeast Two-Hybrid System (Clontech) and a HeLa cDNA library. Recombinant, leucine-tryptophan auxotrophic yeast Y190 were grown on SCAD agar plates in the absence (1, 2, 5 and 6) and presence of leucine and tryptophan and X-Gal (3, 4, 7 and 8). (1 and 3) Recombinant yeast carrying the empty vector pGADH with the GAL4 activation domain and the vector pGBT encoding *ankF* fused to the GAL4 binding domain. (2 and 4) Recombinant Y190 carrying the empty vector pGBT and the vector pGADH-vimentin. (5 and 7) Recombinant Y190 carrying the vector p53pBD and vector pSV40-pAD-gal4 containing the large T-antigen of the SV40 virus fused to the Gal4 activation domain as positive control interaction partners. (6 and 8) Recombinant Y190 carrying the vector pGBT9-ankF and the vector pGADH-vimentin. The image is representative of three independent experiments. **(B, C)** Stably-transfected HeLa-AnkF cells (HeLa-pWHE644/655-AnkF), harboring a doxycycline-inducible AnkF-expression system, were incubated without or with 1 µg/ml doxycycline for the indicated durations. **(B)** Cell lysates were separated by SDS-PAGE, transferred to a PVDF membrane, and probed with antibodies against AnkF, vimentin and actin as loading control. A representative image of three independent experiments is shown. **(C)** Total isolated RNA was reverse-transcribed using SuperScript II reverse transcriptase according to the manufacturer’s protocol and a qRT-PCR was performed with primers specific for *vimentin* (936 and 937) and *actin* (827 and 828) as a housekeeping gene. Vimentin was normalized to actin and is depicted as fold change compared to non-induced cells. **(D)** CHO-FcR cells were transiently transfected with plasmids pCMV-HA-vimentin (red) together with pGFP (green) alone (upper panel) or together with pEGFP-AnkF (lower panel). 24 h post-transfection, cells were fixed and HA-vimentin was stained with specific antibodies by indirect immunofluorescence (red). Nuclei were stained with DAPI (blue). Transient expression of HA- and GFP-containing proteins was visualized by epifluorescence microscopy. Images are representative of three independent experiments.

To learn how AnkF might alter the CCV to allow bacterial replication, we performed a yeast two-hybrid assay to identify potential host cell interaction partners. Using a HeLa genomic library, the intermediate filament vimentin was found to bind AnkF. Performing a LacZ filter-lift assay, X-Gal dye precipitation by interaction of AnkF and vimentin exceeded the signal obtained with the positive control (p53 and t-antigen (**Figure 7A**). Next, we determined how the AnkF-vimentin interaction might influence vimentin function. Modification of vimentin by bacterial products has been reported before. Thus, *Mycobacterium tuberculosis* down-regulates vimentin expression (Garg et al., 2006; Mahesh et al., 2016). In order to test whether vimentin was modified at the level of gene expression or steady-state protein in the presence of ectopic AnkF, quantification of vimentin expression at both mRNA and protein levels was performed. Stable HeLa-*ankF* cell lines harboring a doxycycline-inducible AnkF expression system (Berens et al., 2015) were used to quantify vimentin mRNA expression by RT-PCR and protein synthesis by immunoblot analysis. While we detected increasing protein levels of AnkF starting from 4 h post-induction, we did not observe an influence of AnkF on the vimentin protein level or stability (**Figure 7B**). No change in mRNA expression occurred in the presence of expressed AnkF (**Figure 7C**). Thus, AnkF does not influence vimentin transcriptionally or translationally.

Next, we performed co-localization studies. Over-expression of GFP-tagged AnkF and HA-tagged vimentin in CHO cells showed a clear co-localization (**Figure 7D**). Moreover the co-expression alters the filamentous appearance of HA-vimentin into a punctate-like localization (**Figure 7D**), suggesting that AnkF might alter vimentin assembly and localization within the cell.

### Vimentin Is Recruited to the CCV

**Figure 8:**
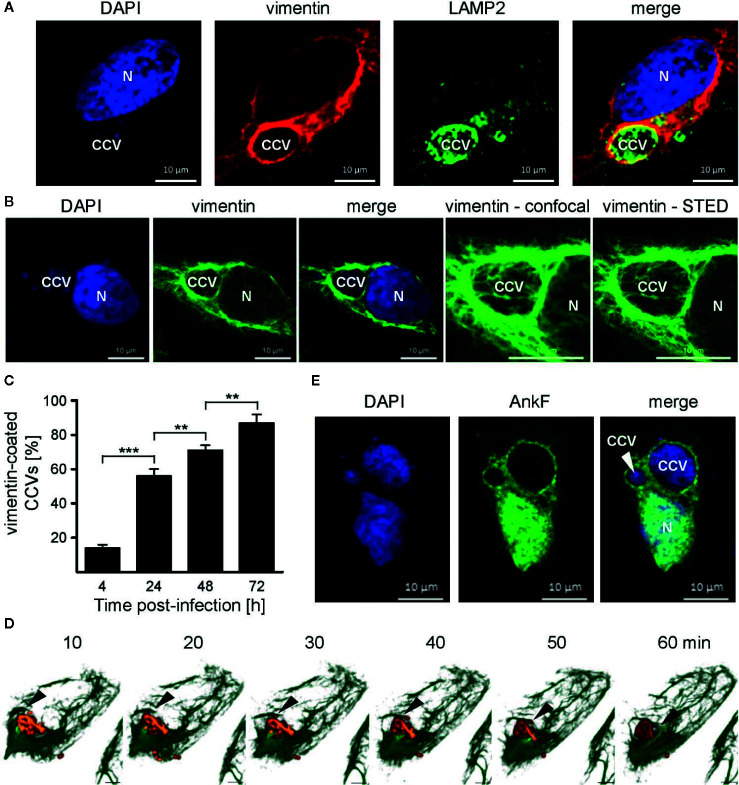
Vimentin and AnkF associate with the CCV. **(A)** HeLa cells were infected with *C. burnetii* at an MOI of 50 for 24 h. Cells were fixed and stained with vimentin- and LAMP2-specific antibodies by indirect immunofluorescence (red and green, respectively). Nuclei and bacterial DNA were stained with DAPI (blue). Cells were visualized using LSM. **(B)** Infected cells were fixed and stained with a vimentin-specific antibody by indirect immunofluorescence (green). Nuclei and bacterial DNA were stained with DAPI (blue). Cells were visualized using LSM or Stimulated Emission Depletion (STED) high-resolution laser scanning fluorescence microscopy to highlight the close association of vimentin with the CCV. **(C)** HeLa cells were infected with *C. burnetii* at an MOI of 50 for up to 72 h. At the time points indicated, cells were fixed and vimentin and *C. burnetii* were stained with specific antibodies by indirect immunofluorescence. Using LSM, 100 cells were counted per each of three independent experiments for association of vimentin with the CCV. Error bars indicated ± SD. **p < 0.01, ***p < 0.001. **(D)** Stable U2-OS-rseGFP-vimentin cells were infected with recombinant, inducible td-Tomato-expressing *C. burnetii* in the presence of 2 mM IPTG at an MOI of 100. At 72 h post infection, live-cell imaging was performed using Spinning Disc Confocal Microscopy. Z-stack images were acquired in a 15-μm range in 0.2-μm intervals every 10 min during a time period of 1 h. 3D re-construction was performed using ZEN software (Carl Zeiss AG) to visualize rseGFP-vimentin (green) and *C. burnetii*-tdTcc (red). Black arrows indicate the growing tip of vimentin filaments on the CCV. Scale bar: 5 μm. **(E)** HeLa cells were infected with *C. burnetii* at an MOI of 50. After 6 days, cells were transfected with pcDNA-AnkF. 24 h post-transfection, the cells were fixed and stained with an anti-AnkF-serum (green). Nuclei and bacterial DNA were stained with DAPI (blue). A representative LSM image from three independent experiments is shown.

Physiologically, vimentin is involved in intracellular trafficking events (Styers et al., 2005). Depending on the intracellular conditions and the stresses involved, vimentin can either be flexible, but also of stabilizing nature (Janmey et al., 1991). Thus, vimentin provides a stabilizing scaffold for *C. trachomatis* inclusions during infection (Kumar and Valdivia, 2008). Additionally, siRNA-mediated knock-down of vimentin was shown to reduce the number of CCVs in host cells (McDonough et al., 2013). This led us to investigate whether vimentin is recruited to the CCV during infection. Thus, HeLa cells were infected with *C. burnetii* and localization of endogenous vimentin and LAMP2 was visualized by immunofluorescence. At 24 h post-infection, vimentin recruitment to the CCV was visible, as judged by co-localization with LAMP2 (**Figure 8A**). In order to underline the close association of vimentin filaments around the CCV, STED-LSM was performed, demonstrating the close localization of vimentin around the CCV (**Figure 8B**). We quantified the acquisition of vimentin at the CCV and demonstrate that vimentin was recruited to the CCV in a time-dependent manner (**Figure 8C**). The recruitment of vimentin to the CCV correlated with CCV growth, suggesting that association of vimentin with the CCV depends on bacterial replication. However, vimentin filaments are highly dynamic. Thus, analyzing vimentin in fixed cells might be prone to artifacts. Therefore, we infected U2OS-rseGFP-vimentin cells (Ratz et al., 2015) with tdTomato-*C burnetii* and tracked vimentin recruitment to the CCV by live-cell-imaging. **Figure 8D** depicts polymerization of vimentin fibers around the surface of the CCV within a timeframe of 1 h, confirming recruitment of vimentin to the CCV in living cells.

### AnkF Partially Accumulates Around the CCV

In the next step, we investigated whether AnkF, like vimentin, is localized at the CCV. In uninfected cells, GFP- and HA-tagged AnkF exhibit cytoplasmic and nuclear localization when ectopically expressed in HeLa cells (Rodriguez-Escudero et al., 2016). Here, we determined the subcellular localization of AnkF in *C. burnetii* infected cells. In agreement with previous reports (Rodriguez-Escudero et al., 2016), ectopically expressed AnkF localized in the cytosol and within the nucleus. However, we also detected ectopically expressed AnkF in proximity to the CCV (**Figure 8E**), which led us to hypothesize that AnkF might be involved in the recruitment of vimentin to the CCV.

### Vimentin Is Dispensable for *C. burnetii* Replication

**Figure 9:**
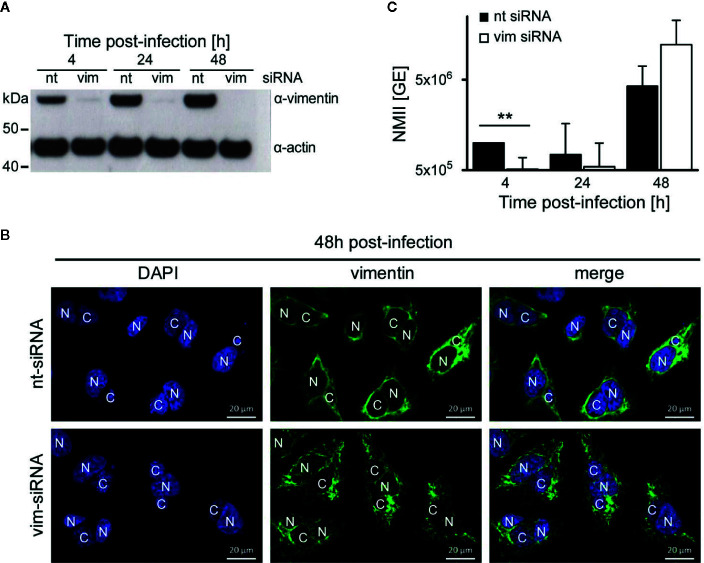
Vimentin is dispensable for *C. burnetii* replication. **(A–C)** HeLa cells were transfected with 50 nM non-targeting (nt) or vimentin-targeting (vim) siRNA. After 24 h post-transfection, cells were infected with *C. burnetii* (WT) at a MOI of 50. **(A)** At the time points post-infection indicated, total cell lysates were separated by SDS-PAGE and analyzed by immunoblot analysis using antibodies against vimentin and actin as loading control. The immune blot is representative of four independent experiments. **(B)** 48 h post-infection, cells were fixed and stained with an anti-vimentin antibody (green). Nuclei and bacterial DNA were stained with DAPI (blue). Images are representative of two independent experiments. N: nucleus, C: *C. burnetii*-containing vacuole. **(C)** At the time-points indicated, cell lysates were prepared by osmotic lysis. Bacteria, isolated by differential centrifugation, were used for preparation of genomic DNA (gDNA). Absolute quantification of bacterial genomic equivalents was performed by quantitative real-time PCR with primers specific for genomic *IS1111* sequences. gDNA, prepared from axenically-grown *C. burnetii* cultures served as standard for genomic equivalents. Error bars represent mean standard deviations of four independent experiments. **p < 0.01.

While we have demonstrated that AnkF is important for efficient intracellular replication, the role of vimentin for bacterial progeny was uncertain. Vimentin is involved in host cell invasion of pathogenic bacteria and supports bacterial replication by providing stability for the bacteria-containing vacuole (Janmey, 1991; Mak and Bruggemann, 2016). To determine the role of vimentin in the host-pathogen*-*interaction, a siRNA-mediated knock-down of vimentin was conducted in *C. burnetii*-infected HeLa cells. An efficient knock-down of vimentin was detected by immunoblot analysis (**Figure 9A**). Our immunofluorescence analysis, in contrast, revealed the presence of smaller vimentin fragments (**Figure 9B**). These fragments might represent soluble tetrameric vimentin (Soellner et al., 1985), which lack the stabilizing activity of filamentous vimentin. Thus, siRNA-mediated knock-down of vimentin allows to determine the role of stabilizing vimentin for *C. burnetii* replication. The knock-down of vimentin reduced the number of *C. burnetii* at 4 h post-infection (**Figure 9C**). At 24 and 48 h post-infection, the absence of vimentin did not seem to influence bacterial replication. These results suggest that the stabilizing function of vimentin is dispensable for intracellular replication of *C. burnetii*.

### Other CCV-Associated Structural Components Are Not Influenced by AnkF Expression

**Figure 10:**
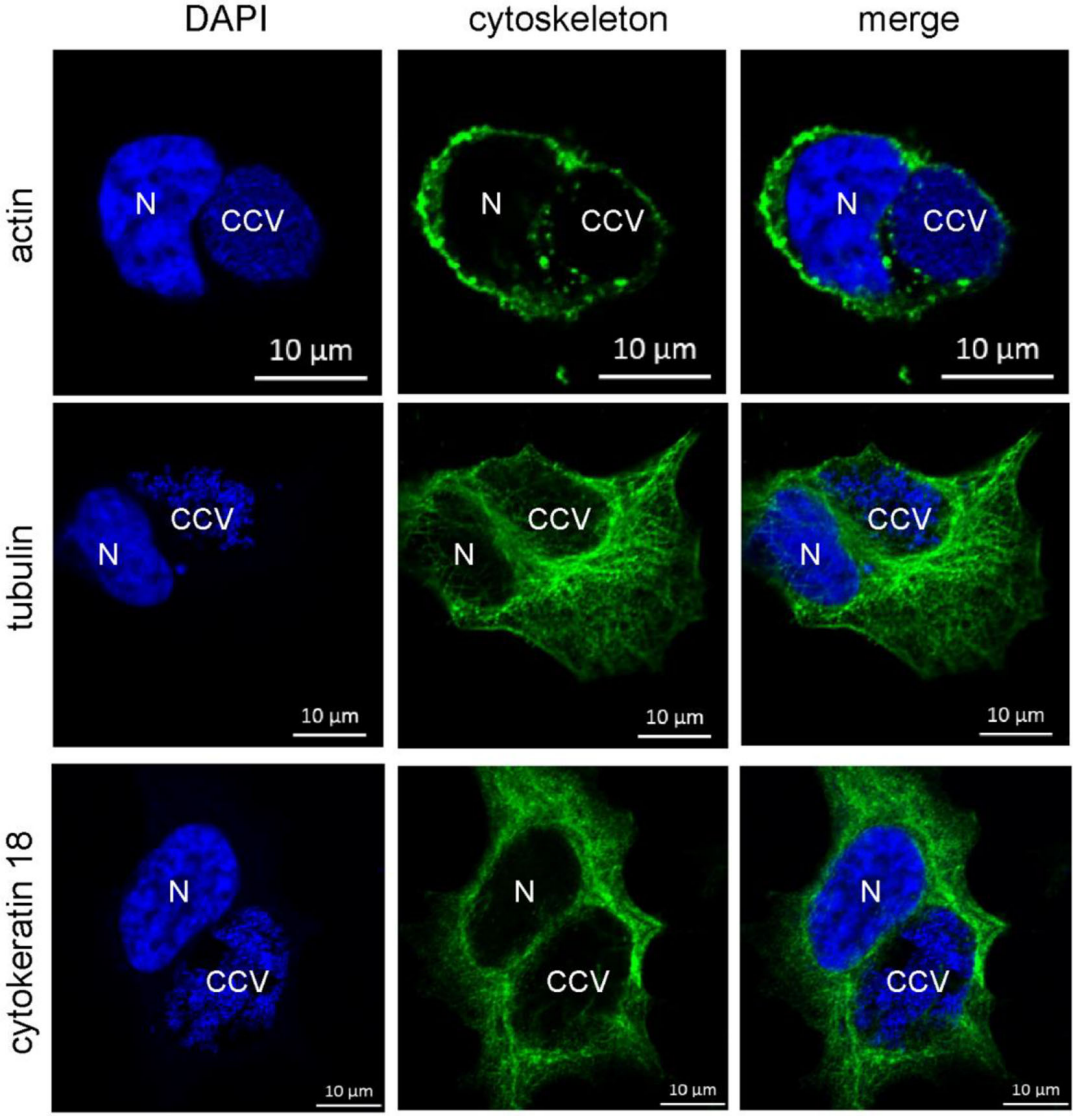
Cytoskeletal filaments decorate the CCV. HeLa cells were infected with *C. burnetii* at an MOI of 50. At 72 h post-infection, cells were fixed and stained with Phallotoxin-647 for actin, or with tubulin- and cytokeratin 18-specific antibodies by indirect immunofluorescence (green). Nuclei and bacterial DNA were stained with DAPI (blue). Cells were visualized using LSM. N: Nucleus. CCV: *C. burnetii*-containing vacuole.

However, it might be possible that other intermediate filaments, microfilaments or microtubules compensate for the lack of vimentin. Indeed, cytokeratin 8 and 18 as well as actin act in concert with vimentin to ensure stability of the *C. trachomatis* inclusion (Kumar and Valdivia, 2008). Furthermore, actin was shown to associate with the CCV, and network disruption might result in smaller CCVs (Aguilera et al., 2009; Miller et al., 2018). It was proposed that actin stabilizes the CCV (Colonne et al., 2016) and participates in vesicular trafficking events (Aguilera et al., 2009; Miller et al., 2018). In order to elucidate the association of microfilaments, microtubules, and cytokeratins with bacterial compartment, immunofluorescence of *C. burnetii*-infected HeLa cells was performed. At 72 h post-infection, actin forms a punctate-like pattern around the bacterial compartment, whereas tubulin and cytokeratin 18 associate around the bacterial compartment in a filamentous pattern (**Figure 10**). Thus, these cytoskeletal components might be involved in stabilizing the CCV and might compensate for vimentin deficiency.

**Figure 11:**
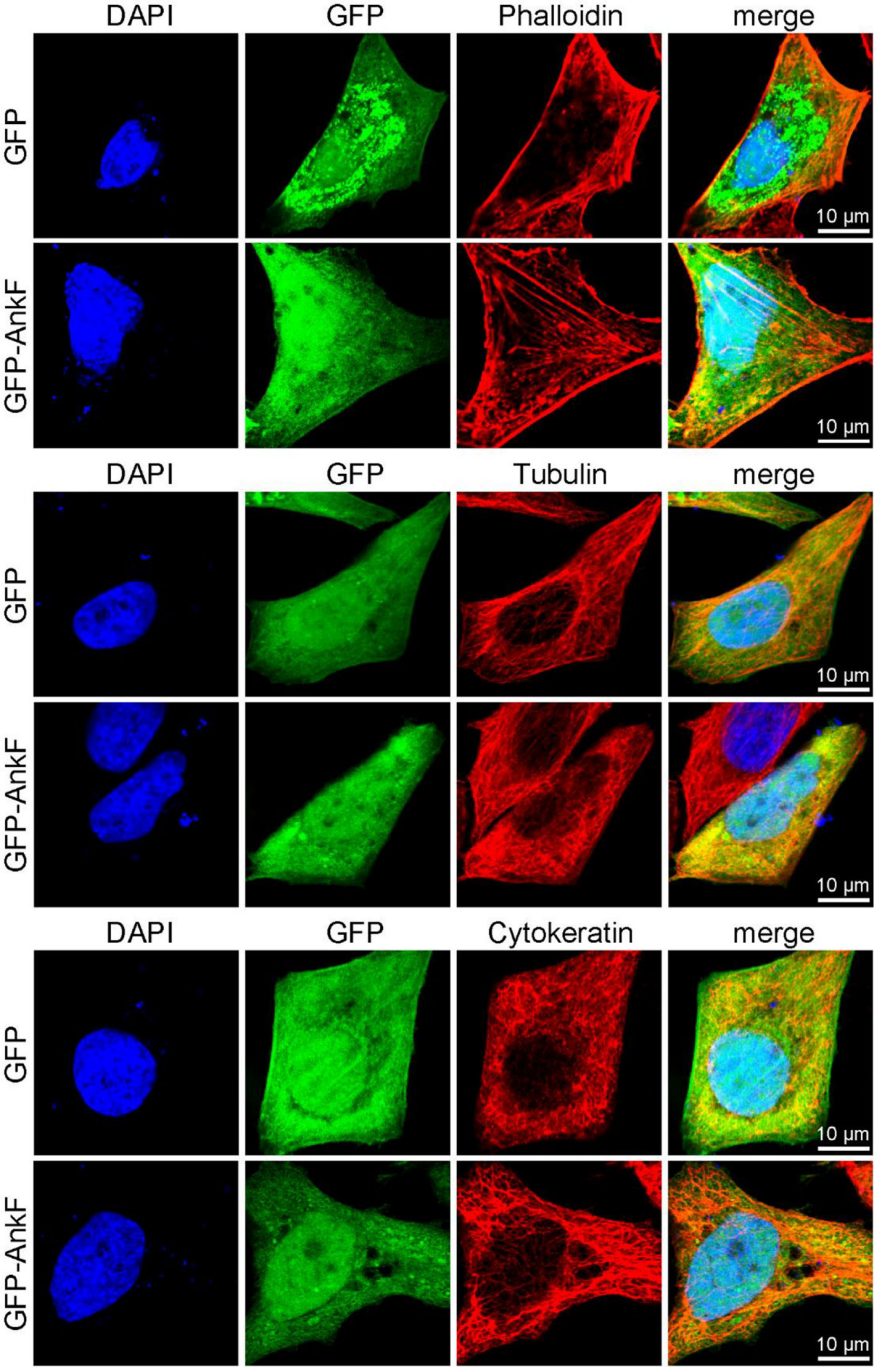
Cytoskeletal filaments are not modified by AnkF expression. HeLa cells were transiently transfected with pGFP alone or with pEGFP-AnkF (green). 24 h post-transfection, cells were fixed and stained with Phalloidin-Alexa647 for actin, or with tubulin- and cytokeratin 18-specific antibodies by indirect immunofluorescence (red). Nuclei and bacterial DNA were stained with DAPI (blue). Cells were visualized using LSM. Representative images from two independent experiments are shown.

In order to elucidate whether AnkF expression might also influence the subcellular localization of these cytoskeletal components, we ectopically expressed GFP-AnkF or GFP as control in HeLa cells and analyzed the structure of actin, tubulin, and cytokeratin by immunofluorescence. As demonstrated in **Figure 11**, the expression of GFP-AnkF did not disturb the localization of these cytoskeletal components, suggesting that AnkF might specifically modify vimentin localization.

## Discussion

The *C. burnetii* protein AnkF was shown to be injected into the host cell in a T4SS-dependent manner (Pan et al., 2008). The chaperone IcmS, which is required for the translocation of a subset of *C. burnetii* T4SS effector proteins (Larson et al., 2019), however, is dispensable for AnkF translocation (Voth et al., 2009). Where AnkF localizes within the host cell is still unclear. Ectopically expressed AnkF was found in the host cell cytoplasm, the nucleus (Rodriguez-Escudero et al., 2016) and in the vicinity of the CCV (**Figure 8E**). This multiple and diverse localization may explain why our attempts to localize translocated AnkF have failed so far. Another reason might be the short intracellular half-life of AnkF (Pan et al., 2008).

By comparing five different isolates, AnkF was identified as a highly conserved effector protein (Voth et al., 2009; Larson et al., 2016). Our analysis of 52 isolates confirmed this assumption (**Figure 1**). This is an interesting observation, as the majority of genes encoding for effector proteins were characterized by considerable heterogeneity between different *C. burnetii* isolates (Bisle et al., 2016; Larson et al., 2016). Conserved effector proteins might therefore be important virulence determinants. Indeed, the insertion of a transposon in the *ankF* gene at position 507 results in a markedly reduced ability to replicate intracellularly (**Figures 3B–E**, **4A, C**). In a previous publication, a different transposon mutant carrying a transposon insertion at base pair 291 in *ankF* showed no effect on bacterial intracellular replication (Martinez et al., 2014). However, our experiments indicate that this transposon mutant is not clonal and contains wild-type AnkF (data not shown), which might explain the lack of phenotype. Importantly, while we observed an inability of the *ankF* transposon mutant to replicate intracellularly, we only noted a moderate reduction in intracellular replication of the *ankF* deletion mutant (**Figures 6A, B**). The underlying reason for this difference might be transposon-mediated off-target effects in the genomic neighborhood of *ankF*. Indeed, a transposon insertion within *cbu0446*, a gene flanking *ankF* (*cbu0447*), was shown to cause a strong replication defect (Martinez et al., 2014). Further experiments are required to elucidate whether AnkF and CBU0446 are influencing each other.

While the activity of AnkF is still unclear, we showed that it binds vimentin (**Figure 7A**) and modulates vimentin filamentous assembly (**Figure 7D**). We propose that AnkF recruits vimentin to the CCV, as AnkF partially localizes to the CCV (**Figure 8E**) and the timing of recruitment of vimentin to the CCV (**Figure 8C**) correlates with effector protein translocation, which starts not earlier than 8 h and peaks around 24 h post-infection (Newton et al., 2013). While the domain important for binding to vimentin was not identified in this study, it is tempting to speculate that the ankyrin repeat domains in AnkF (Voth et al., 2009) might be involved. Ankyrin repeats are important protein-protein interaction domains (Mosavi et al., 2004), and the protein Ankyrin 1 was shown to bind to vimentin and mediate the association of vimentin with erythrocyte membranes (Georgatos and Marchesi, 1985).

Vimentin belongs to the class of intermediate filaments (IF), which are major elements of the cytoskeleton (Herrmann et al., 2009). Assembly and disassembly of vimentin filaments is mediated by post-translational modifications (Eriksson et al., 2004). In comparison to actin and tubulin, vimentin fibers are less rigid but more resistant to tensile forces (Janmey, 1991). Vimentin plays an important role in stabilizing cellular organelles and in organelle-positioning within the cytoplasm (Minin and Moldaver, 2008). In addition to its role as a cytosolic protein, vimentin has been reported to be cell surface-located and extracellularly localized (Mor-Vaknin et al., 2003). Vimentin is also involved in bacterial infections. Thus, vimentin and keratin 18 bind to the *Shigella flexneri* type III secretion system (T3SS) translocon pore protein IpaC. This interaction facilitates the docking of the bacteria to the host cell and enables effector protein translocation, which is crucial for virulence (Russo et al., 2016). Vimentin not only influences the translocation of effector proteins, it also influences bacterial invasion, the stability of the replicative niche and innate immune responses (Mak and Bruggemann, 2016). Thus, surface-located vimentin mediates invasion of Group B *streptococci*, *Listeria monocytogenes*, *Escherichia coli* K1 and *Propionibacterium acnes* (Mak et al., 2012; Huang et al., 2016; Bastounis et al., 2018; Ghosh et al., 2018; Deng et al., 2019). Vimentin is also important for cell entry of several viruses, including SARS-CoV (Mak and Bruggemann, 2016; Yu et al., 2016). Our data suggest that vimentin might also be involved in the entry of *C. burnetii* in non-phagocytic cells (**Figure 9C**). Whether vimentin plays a role in *C. burnetii* invasion in phagocytic cells requires testing. Complement receptor 3 and α_v_β_3_ integrin are involved in the phagocytosis of *C. burnetii* by phagocytic cells (Capo et al., 1999). Vimentin interacts with β_3_ integrin, which is proposed to increase β_3_ integrin clustering at the plasma membrane and to support β_3_ integrin-ligand binding (Kim et al., 2016). This makes it likely that vimentin participates in *C. burnetii* uptake into phagocytic cells.

Moreover, expression of vimentin influences immune signaling, including activation of NF-κB (Mor-Vaknin et al., 2013) and the MAP kinase ERK1/2. Thus, vimentin promotes ERK1/2 signaling during *S.* *enterica* infection (Murli et al., 2001). Similarly, vimentin-induced ERK1/2 activation facilitates an *A. phagocytophilum* infection (Sukumaran et al., 2011). Likewise, a *C. burnetii* infection leads to the activation of the MAP kinase ERK1/2 (Boucherit et al., 2012; Graham et al., 2013), which is required for the anti-apoptotic activity of *C. burnetii* (Voth and Heinzen, 2009). Whether ERK1/2 activation during *C. burnetii* infection is mediated by vimentin is unknown. It is possible that vimentin is not only required as scaffolding for the CCV, but also as an inducer of immune signaling.

In addition, vimentin stabilizes or positions bacteria-containing vacuoles (Guignot and Servin, 2008; Kumar and Valdivia, 2008; Mak and Bruggemann, 2016; Truchan et al., 2016). Thus, vimentin contributes to the stability of the *C. trachomatis* inclusion. However, while the absence of vimentin influences the stability and morphology of the inclusion, it does not seem to affect bacterial replication (Kumar and Valdivia, 2008). The *Anaplasma phagocytophilum*-containing vacuole is also encased by vimentin (Truchan et al., 2016). In contrast to infection with *C. trachomatis* (Kumar and Valdivia, 2008) and *C. burnetii* (**Figures 7B, C**), the infection with *A. phagocytophilum* resulted in increased vimentin expression (Truchan et al., 2016). Pharmacologic inhibition of soluble vimentin did not reduce bacterial replication when administered to *A. phagocytophilum*-infected cells (Truchan et al., 2016). Similarly, siRNA-mediated knock-down of vimentin did not influence *Salmonella* Typhimurium replication (Guignot and Servin, 2008). Thus, our observation that the lack of vimentin does not influence bacterial replication (**Figure 9C**) is in line with previous publications (Guignot and Servin, 2008; Kumar and Valdivia, 2008; Truchan et al., 2016). The reason why vimentin seems to be dispensable for bacterial replication is elusive. One explanation might be functional redundancy. Thus, other intermediate filaments or microfilaments might be able to compensate for the lack of vimentin. Of note, the recruitment of vimentin to the *C. trachomatis* inclusion is dependent on actin microfilaments, which also decorate the inclusion. In addition, the intermediate filaments cytokeratin 8 and 18 were also recruited to the inclusion providing stability (Kumar and Valdivia, 2008). Our data demonstrate that microtubules and the intermediate filament cytokeratin 18 associate around the bacteria (**Figure 10**), making it possible that microtubules and other intermediate filaments compensate for the vimentin knock-down. The functional redundancy might explain the lack of a replication-defect in the absence of vimentin (**Figure 9C**) (Guignot and Servin, 2008; Kumar and Valdivia, 2008; Truchan et al., 2016). Furthermore, actin patches surrounded the CCV (**Figure 9**), as reported previously (Miller et al., 2018). Miller and colleagues showed that the lack of actin patches did not affect *C. burnetii* replication. However, actin filaments produced in an Arp2/3-dependent manner were required for vesicular trafficking events, and, thus, for CCV generation (Miller et al., 2018). Based on these data, we suggest that microfilaments and different intermediate filaments, including vimentin and cytokeratin 18, are recruited to the CCV to provide stability; at the same time they provide a platform for fusion and fission events, which allows the replicative CCV to form.

## Data Availability Statement

The original contributions presented in the study are included in the article/supplementary material. Further inquiries can be directed to the corresponding author.

## Author contributions

JP, JS-L, SB, FC, and MÖ performed the experiments and analyzed the data. MB and PAB provided resources, CB analyzed data, and AL conceived the study, obtained funding, supervised the study and drafted the manuscript. All authors contributed to the article and approved the submitted version.

## Conflict of Interest

The authors declare that the research was conducted in the absence of any commercial or financial relationships that could be construed as a potential conflict of interest.
